# Application of endoport-assisted neuroendoscopic techniques in lateral ventricular tumor surgery

**DOI:** 10.3389/fonc.2023.1191399

**Published:** 2023-10-17

**Authors:** Chaolong Yan, Jiannan Mao, Chenbei Yao, Yang Liu, Wei Jin, Huiying Yan

**Affiliations:** ^1^ Department of Neurosurgery, Nanjing Drum Tower Hospital, Affiliated Hospital of Medical School, Nanjing University, Nanjing, China; ^2^ Department of Neurosurgery, Nanjing Drum Tower Hospital, Clinical College of Nanjing Medical University, Nanjing, China

**Keywords:** endoport, neuroendoscopy, lateral ventricular tumor, surgical treatment, prognosis

## Abstract

**Objective:**

The objective of this study was to investigate the clinical experience and therapeutic efficiency of Endoport-assisted neuroendoscopic surgery for resection of lateral ventricular tumors. The key points and application value of this surgical technique were additionally discussed.

**Methods:**

A retrospective analysis was conducted on the clinical and follow-up data of 16 patients who underwent endoport-assisted neuroendoscopic surgery for lateral ventricular tumors at the Department of Neurosurgery, Nanjing Drum Tower Hospital, the Affiliated Hospital of Nanjing University Medical School, between January 2018 and September 2020. The surgical procedures, complications and outcomes were analyzed.

**Results:**

The study included a total of 16 patients (5 males and 11 females) with lateral ventricular tumors, with a mean age of 43.2 years (18-70 years old). The tumors were distributed as follows: 5 cases involved the body of the lateral ventricle, 3 involved the frontal horn and body, 3 involved the occipital horn, 2 involved the trigone, 2 involved the frontal horn, and 1 case involved the occipital horn and body. Perioperative complications were analyzed, revealing 1 case of intraoperative acute epidural hematoma intraoperative and 2 cases of postoperative obstructive hydrocephalus. All complications were promptly managed. Postoperative MRI revealed that 14 cases (88%) achieved total resection, while 2 cases (12%) achieved subtotal resection. During the follow-up of 6-38 months, no recurrence was observed. The patient diagnosed with glioblastoma died 16 months after surgery (GOS=1), while the remaining patients have successfully resumed to normal daily life with a GOS score of 5.

**Conclusion:**

In conclusion, endoport-assisted neuroendoscopic surgery proved to be a minimally invasive and effective technique for resecting lateral ventricular tumors, with acceptable complications. It effectively utilizes the benefits of close observation, comprehensive exposure, and reduced tissue damage. Therefore, endoport-assisted neuroendoscopic surgery is suitable for the resection of lateral ventricular tumors.

## Introduction

The lateral ventricle, is a paired C-shaped structure located deep within the cerebral hemispheres (1, 2). Lateral ventricular tumors, accounting for less than 1% of intracranial neoplasms, mostly exhibit slow growth (3, 4). Early diagnosis and complete resection of these tumors hold the potential for clinical cure. Hence, neurosurgeons managing lateral ventricular tumors must strive for maximal tumor resection while minimizing damage to nerves and blood vessels (5).

In recent years, with advancements in surgical techniques and the adoption of minimally invasive approaches, the endoport-assisted neuroendoscopic technique has emerged as a promising method for resecting lateral ventricular tumors (2, 6, 7). Furthermore, the widespread use of neuro-navigation systems and intraoperative electrophysiological monitoring has contributed to overcoming challenges such as endoscopic hemostasis and the protection of functional areas and neural nuclei. These advancements have resulted in improved efficacy of neuroendoscopy in the treatment of lateral ventricular tumors.

## Materials and methods

### Clinical data collection

A retrospective review was conducted, encompassing clinical and follow-up data from 16 patients who presented with lateral ventricular tumors and subsequently underwent endoport-assisted neuroendoscopic surgery between January 2018 and September 2020 in the department of neurosurgery in Nanjing Drum Tower Hospital, The Affiliated Hospital of Nanjing University Medical School. The research protocol involving human participants was approved by the Ethics Committee at Nanjing Drum Tower Hospital.

Data collection involved a comprehensive assessment of medical records, imaging data, pathological reports, and operation videos for each patient. Demographic information, initial symptoms, tumor location, pathological diagnosis, and the presence of preoperative hydrocephalus were documented. Additionally, complications that occurred during or after operation were carefully recorded for analysis. Follow-up was obtained in the clinic or by telephone and ended in March 2021.

### Surgical equipment

All operations were performed using rigid Karl Storz neuroendoscopy with 0° or 30° optics, 150 mm length and 4 mm diameter (Karl Storz, Tuttlingen, Germany). An endoport with a diameter of 21 mm and length of 7 cm (Vycor Medical Inc., Boca Raton, FL, USA) was selected to handle the lesions in our institute. To facilitate the surgery, a pneumatic holding arm (Karl Storz, Tuttlingen, Germany) was utilized to stabilize the neuroendoscopy, while a snake holding arm was employed to secure the endoport. Additionally, conventional craniotomy instruments and neuroendoscopic auxiliary instruments were used during the surgical procedures.

### Surgical techniques

#### The trans-frontal cortical approach to the frontal horns and the body of the lateral ventricle

**Figure 1:**
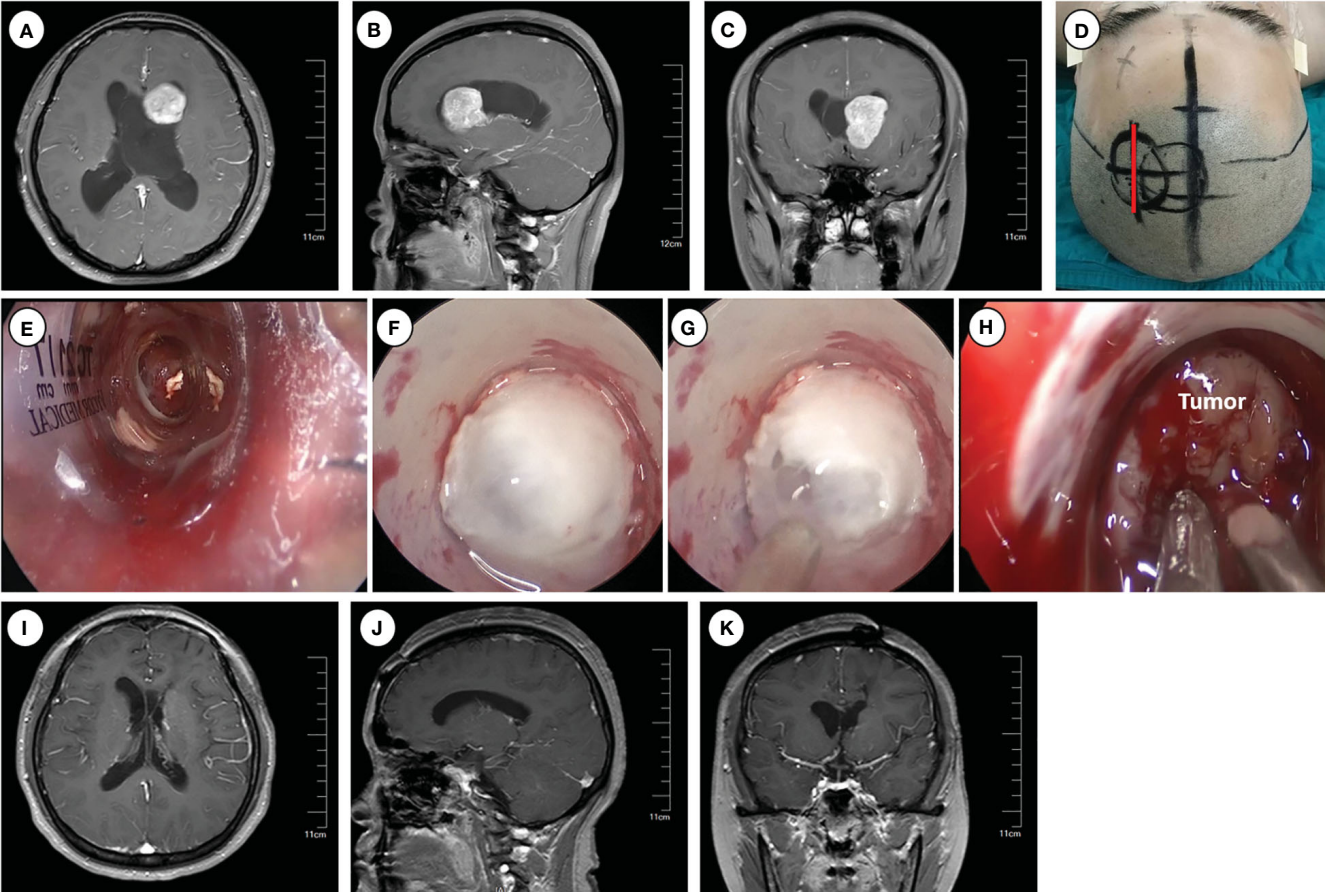
Operative approaches for the frontal horns and the body of the lateral ventricle. **(A–C)** Axial, sagittal and cronal images of preoperative enhanced magnetic resonance imaging. **(D)** Schematic diagram of surgical incision (marked with red line). **(E–H)** Intraoperative pictures for inserting Endoport **(E)**, ventriculostomy to release CSF **(F–G)**, and tumor resection **(H)**. **(I–K)** Axial, sagittal and coronal images of enhanced magnetic resonance imaging at 6 months postoperative.

The trans-frontal cortical approach is an effective surgical route for accessing the frontal horns and bodies of the lateral ventricle, including the anterior part of the third ventricle (**Figure 1**). It is particularly suitable for resecting tumors mainly limited in the frontal horns or the bodies of the lateral ventricle. The patient was placed in the supine position with the head slightly elevated. The head was turned 30° to the contralateral side and secured with a 3-pin Mayfield head holder. Neuro-navigation based on preoperative magnetic resonance imaging (MRI) was employed to guide the anatomical localization. The insertion point for endoport was approximately 3 cm from the midline and 1 cm in front of the coronal suture (Kocher’s point).

Following routine disinfection, a linear incision was made centered on the insertion point. A craniotomy (approximately 3 cm diameter) was performed above the middle frontal gyrus, and the dura was opened with an arc-shaped incision. An ostomy through the middle frontal gyrus was created to insert and secure the endoport using the snake holding arm. Cerebrospinal fluid (CSF) outflow confirmed entry into the lateral ventricle. Tailed cotton strips were used to slowly release CSF while securing a 0° neuro-endoscope into the endoport using the pneumatic holding arm. Gradual identification of the anatomical landmarks of the frontal horn and body of the lateral ventricle was achieved. Under neuroendoscopic visualization, the tumors were carefully observed and removed. An ultrasonic dissector could be utilized for tumors with hard texture. For larger tumors, intratumoral decompression was performed initially to gradually reduce tumor volume. Adjustments in the directions of the endoport and neuro-endoscope may be necessary to identify the tumor boundary and achieve complete tumor removal.

Following tumor resection, bipolar coagulation and hemostatic materials were employed to ensure thorough hemostasis. Special care was taken to protect the surrounding brain tissue and vasculature, particularly the lateral wall of the lateral ventricle, where important nervous nuclei such as the thalamus and caudate nucleus, as well as the colliculus veins, are located. Damage to these structures can significantly impact patient prognosis. Finally, a drainage tube was inserted into the ventricle, and the endoport was removed under endoscopic visualization. Postoperative close monitoring is crucial to detect and manage potential complications.

#### The transtemporal approach to the trigone of the lateral ventricle

**Figure 2:**
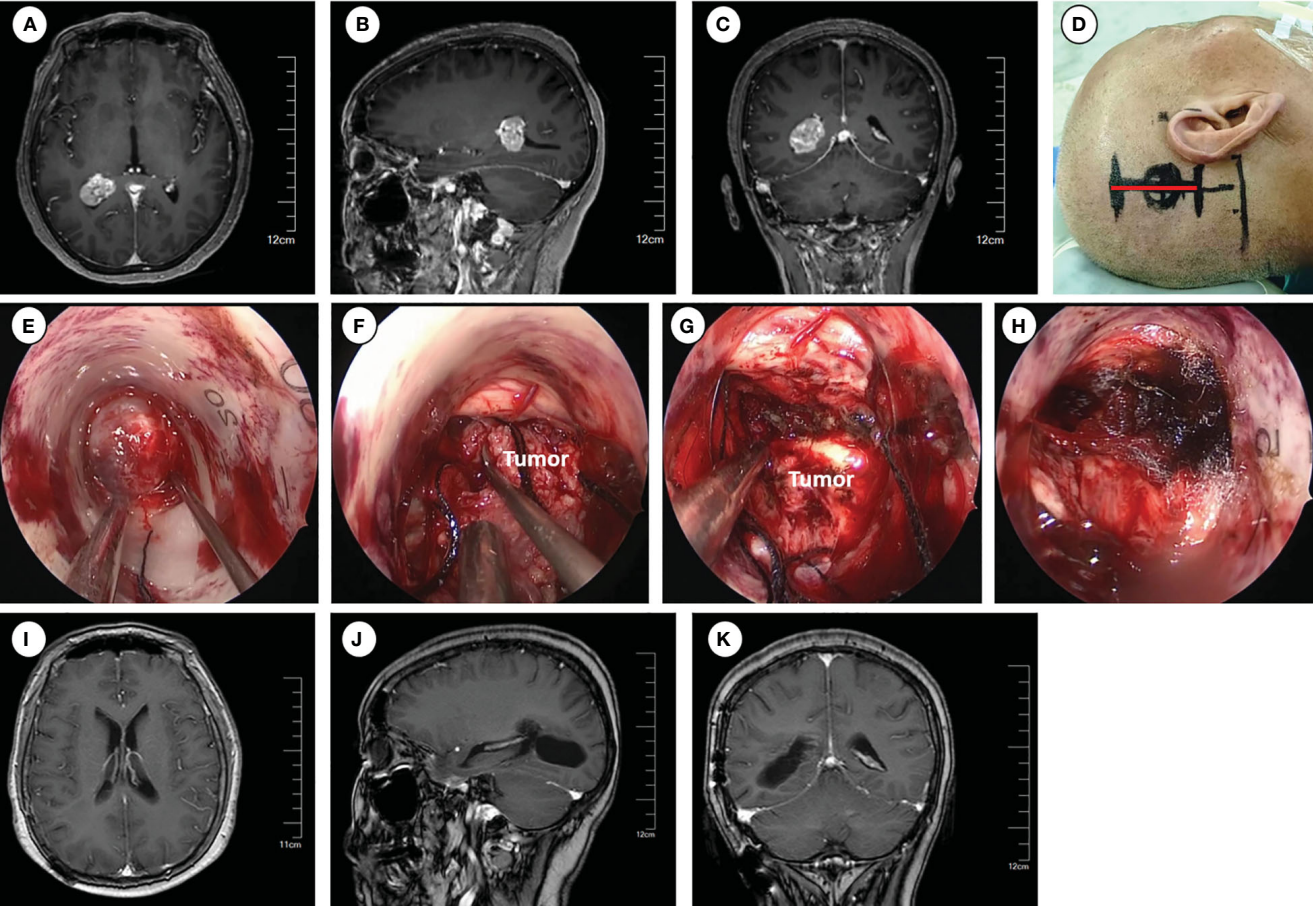
Operative approaches for the trigone of the lateral ventricle. **(A–C)** Axial, sagittal and coronal images of preoperative enhanced magnetic resonance imaging. **(D)** Schematic diagram of surgical incision (marked with red line). **(E–H)** Intraoperative pictures for exposing the tumor **(E)**, tumor removal **(F–G)**, and hemostasis of the surgical field **(H)**. **(I–K)** Axial, sagittal and coronal images of enhanced magnetic resonance imaging at 6 months postoperative.

The trigone, located at the interface between the body of the lateral ventricle and the occipital and temporal horns (**Figure 2**), can be accessed directly using the transtemporal approach. This approach offers a short trajectory and provides direct access to the temporal horn and trigone of the lateral ventricle. It is particularly suitable for removing tumors located in the trigone of the lateral ventricle. The patient was positioned in the lateral decubitus position, with the head fixed using a 3-pin Mayfield head holder and rotated to the contralateral side to optimize the operative field by positioning the zygomatic process uppermost. Neuro-navigation based on preoperative magnetic resonance imaging (MRI) was employed for precise anatomical localization. The insertion point for endoport was approximately 4.5 cm above and behind the external auditory canal.

Following routine disinfection, a linear incision was made centered on the insertion point. The dura was then opened with an arc-shaped incision. The ostomy through the parietal lobe was created to insert and secure the Endoport using the snake holding arm. Once cerebrospinal fluid (CSF) outflow was observed, entry into the trigone of the lateral ventricle was confirmed. Tailed cotton strips were used to slowly release CSF while fixing a 0° neuroendoscopy into the endoport using the pneumatic holding arm. The tumors were carefully observed and removed under neuroendoscopic visualization. The subsequent tumor removal procedures were the same as described previously.

### Clinical efficacy evaluation

All cases underwent a computed tomography (CT) scan within 24 hours post-surgery to rule out postoperative complications such as hemorrhage or hydrocephalus. Plain and enhanced magnetic resonance imaging (MRI) scans three days post-surgery, were performed to assess the extent of surgical resection. The need for further radiotherapy or chemotherapy was determined based on postoperative pathological analysis. Subsequently, follow-ups were conducted through outpatient visits or regular telephone interviews at 1, 3, and 6 months postoperatively, followed by annual follow-ups thereafter. During these follow-ups, tumor recurrence and the patients’ ability to carry out daily activities were assessed and recorded. Clinical outcomes were evaluated with the Glasgow Outcome Scale (GOS): a score of GOS ≥ 4 indicates favorable prognosis, while a score of GOS < 4 indicates poor prognosis.

## Results

### Clinical features and histopathological types of patients with lateral ventricular tumors

A total of 16 patients (5 males, 11 females) who suffered from lateral ventricle tumors and underwent endoport-assisted neuroendoscopic surgery were included in this study. The mean age of the patients was 43.2 years, ranging from 18 to 70 years. Upon admission, the patients presented with various initial symptoms, including headache (8 cases), dizziness (3 cases), unconsciousness (1 cases), facial numbness (1 cases) and no apparent symptom (3 cases). The distribution of tumor locations among the cases was as follows: 7 cases in the left and 9 cases in the right, involved the lateral ventricle body (5 cases), frontal horn and body (3 cases), occipital horn (3 cases), trigone (2 cases), frontal horn (2 cases) and occipital horn and body (1 case). Additionally, 9 cases (56.25%) presented with preoperative obstructive hydrocephalus.

All the tumors were successfully removed using endoport-assisted neuroendoscopic surgery, and all the resected specimens were sent to the neuropathology department for the detailed histopathological examination. The postoperative histopathological results were as follows: central neurocytoma (WHO II) in 5 cases, meningioma (WHO I) in 5 cases, diffuse astrocytoma (WHO II) in 2 cases, astrocytoma (WHO I) in 1 case, glioblastoma (WHO IV) in 1 case, ependymoma (WHO II) in 1 case, and colloid cyst in 1 case. A summary of the clinical features and histopathological types of the patients is presented in **Table 1**.

**Table 1:** Clinical features, histopathological types and follow-up results of patients with lateral ventricular tumors.

No.	Gender	Age	Initial symptom	Location	Side	Hydrocephalus	Histopathology*	Surgical complication	Resection	Follow-up (Months)	GOS score
01	Female	25	Headache	Frontal horn, body	Right	Yes	Central neurocytoma (WHO II)	No	Total	38	5
02	Male	31	Asymptom	Frontal horn	Left	No	Central neurocytoma (WHO II)	No	Total	36	5
03	Female	23	Headache	Body	Right	Yes	Central neurocytoma (WHO II)	No	Total	36	5
04	Female	18	Headache	Body	Left	Yes	Astrocytoma (WHO I)	Acute epidural hematoma	Total	35	5
05	Female	37	Headache	Frontal horn, body	Left	Yes	Glioblastoma (WHO IV)	Hydrocephalus, external ventricular drainage	Subtotal	16	1
06	Male	70	Unconsciousness	Frontal horn, body	Left	Yes	Diffuse astrocytoma (WHO II)	Transient hemiplegia, improved after 3 weeks	Subtotal	26	5
07	Female	60	Dizziness	Trigone	Right	No	Meningioma (WHO I)	No	Total	23	5
08	Female	65	Dizziness	Trigone	Left	No	Ependymoma (WHO II)	No	Total	19	5
09	Male	64	Facial numbness	Body	Right	No	Diffuse astrocytoma (WHO II)	No	Total	18	5
10	Male	59	Asymptom	Frontal horn	Right	No	Colloid cyst	No	Total	18	5
11	Female	36	Headache	Occipital horn	Right	Yes	Meningioma (WHO I)	No	Total	9	5
12	Female	20	Headache	Occipital horn, body	Right	No	Meningioma (WHO I)	No	Total	7	5
13	Female	62	Headache	Occipital horn	Right	Yes	Meningioma (WHO I)	No	Total	7	5
14	Female	25	Headache	Body	Left	Yes	Central neurocytoma (WHO II)	No	Total	6	5
15	Male	34	Dizziness	Body	Right	Yes	Central neurocytoma (WHO II)	Hydrocephalus, ventriculo-peritoneal shunt	Total	8	5
16	Female	63	Asymptom	Occipital horn	Left	No	Meningioma (WHO I)	No	Total	11	5

*2016 WHO Classification of CNS tumors.

### Perioperative complications

Among the patients, a total of three cases experienced complications either during or after the surgery: 1 case occurred intraoperative and 2 cases occurred postoperative. During the operation, one patient developed frontal acute epidural hematoma, which was promptly addressed, without long-term adverse consequences. Postoperatively, two patients developed obstructive hydrocephalus following operation. One case was successfully alleviated through external ventricular drainage, while the other case required the implementation of a ventriculoperitoneal shunt to achieve relief. Otherwise, one patient experienced transient hemiplegia, which improved after three weeks of active treatment.

### Clinical prognosis

All patients underwent regular follow-up at the outpatient clinic after discharge. The average duration of follow-up was 19.56 months, ranging from 6 to 38 months. The follow-up results revealed that 14 cases (88%) achieved total resection of tumors without residual or recurrence during the follow-up period. In the remaining 2 cases (12%), the resection was considered subtotal. Unfortunately, one of these cases was pathologically diagnosed as glioblastoma (WHO IV) and regrettably passed away 16 months after the operation. The other case was diagnosed with diffuse astrocytoma (WHO II) and is currently undergoing long-term survival with the tumor under standardized treatment in the field of oncology. Based on the long-term follow-up regarding patients’ daily living activities, 15 cases exhibited a favorable prognosis, as indicated by a Glasgow Outcome Scale (GOS) score of 5. Only one case diagnosed with glioblastoma had a poor prognosis, with a GOS score of 1 (**Table 1**).

## Discussion

The occurrence of mass lesions in the lateral ventricle is relatively rare, but it poses significant technical challenges due to its deep location and proximity to vital anatomical structures (8). Lateral ventricular tumors are typically slow-growing and often diagnosed until they reach a large size or cause obstructive hydrocephalus (2). Surgical removal remains the preferred treatment method for these tumors due to the lack of effective drug treatments, and complete surgical resection can lead to a favorable prognosis (9, 10). Thus, surgeons must carefully consider various factors when selecting the appropriate surgical approach for each patient, including achieving effective and complete tumor resection from optimal angles, minimizing disruption and retraction of normal brain tissues, and early exposure of vital anatomical structures. In our study, we performed 16 cases of lateral ventricular tumor resection using endoport-assisted neuroendoscopic surgery. The total resection rate was 88%, which was similar to previous literature reports (**Table 2**) (11–16). There has been no case of recurrence in the follow-up period.

**Table 2:** List of Publications Reporting Resection of Lateral Ventricular Tumors with or without Neuroendoscopy.

Reference	Number of tumors	Population (Adult/Pediatric)	Surgical methods	Gross-Total Resection (%)	Histology	Complications	Recurrent (%)
Vincenzo et al., 2005 (11)	72	Both	Without endoscopy	82% (59/72)	Anaplastic astrocytoma, glioblastoma, meningioma, ependymoma, pilocytic astrocytoma, SEGA, subependymoma, central neurocytoma, choroid plexus papilloma, choroid plexus carcinoma, choroid plexus cysts, ganglioglioma, ganglioglioneurocytoma, PNET, metastases	27.8% (20/72, 4 deep venous thrombosis, 2 respiratory distress, 3 hydrocephalus, 2 extradural hematoma, 5 small intracerebral hematoma, 4 subdural hygroma)	N/A
Jo et al., 2011 (12)	10	Adult	Endoscopy for excision	50% (5/10)	Cavernous angioma, metastasis, meningioma, paragonimus westermani, anaplastic oligodendroglioma, central neurocytoma, brain abscess, glioblastoma	N/A	N/A
Eveline et al., 2016 (13)	12	Pediatric	Endoscopy for excision	92% (11/12)	SEGA, anaplastic ependymoma, neuroepithelial tumor, ependymoma, NGGCT and pilocytic astrocytoma	8.3% (1/12, 1 ICP transitory increase)	2/12 (17%)
Chandrashekhar et al., 2021 (14)	7	N/A	Endoscopy for excision	N/A	Colloid cysts, low-grade glioma, neurocysticercosis	N/A	N/A
	31	N/A	Endoscopy-assisted excision	N/A	Colloid cysts, neurocytomas, and epidermoid cysts	N/A	N/A
Xie et al., 2021 (15)	7	Both	Endoscopy for excision	100%	SEGA, central neuroblastoma, central neuroblastoma, ependymoma, meningioma and metastatic adenocarcinoma	28.6% (2/7, 1 hematoma, 1 visual defect)	0
Suresh et al., 2023 (16)	26	Both	Endoscopy for excision	69% (18/26)	Central neurocytoma, high−grade glioma, ependymoma, colloid cysts, pilocytic astrocytoma, SEGA, PNET	30% (8/28, 4 seizures, 2 transient hemiparesis, 2 visual impairment, 2 mild disturbance in memory, 2 subdural hygroma)	N/A
Current study	16	Adult	Endoscopy for excision	88% (14/16)	central neurocytoma, meningioma, diffuse astrocytoma, astrocytoma, glioblastoma, ependymoma, and colloid cyst	18.8 % (3/16, 1 hematoma, 2 obstructive hydrocephalus)	0

N/A, not applicable; SEGA, subependymal giant cell astrocytoma; NGGCT, nongerminomatous germ cell tumors; PNET, primitive neuroectodermal tumor.

### Application of endoscope in intraventricular tumor surgery

Currently, surgical resection remains the primary treatment method for lateral ventricular tumors. The traditional surgical strategy primarily involves tumor removal by using a microscope through craniotomy. In the majority of cases, cortical incisions or corpus callosotomy are required during the surgery, which inevitably results in damage to normal brain tissue. Moreover, due to the limited field of view provided by the tubular vision of the microscope and the deep location of the tumor, larger incisions are often required to achieve better tumor visualization. In recent years, with advancements in surgical techniques and the evolving concept of minimally invasive surgery, the use of endoscopes in brain surgery increased greatly, from simple fenestrations for obstructive hydrocephalus and arachnoid cysts initially, to biopsy even removing complex tumors from the deeper areas of the brain gradually (5, 16–20). Compared to traditional microscopic surgery, a rigid endoscope allows for access into ventricles, providing superior magnification and illumination for enhanced inspection of regional anatomy and precise lesion dissection (12). Moreover, combined with angle endoscopy enables close observation and access of the hidden angles inside the ventricles to coagulate or clip of vascular pedicle of the tumor (21). The extensive observation range of neuroendoscopy significantly reduces the need for brain tissue traction compared to operating under a microscope, thus better protecting surrounding normal tissue. However, the endoscopic surgery also meets significant limitations, such as the thermal damage of endoscope itself, the operational damage to the blind areas behind the endoscope’s field of view and the limitations of bimanual microsurgical techniques for tumor resection or hemostasis (16).

### Application of Endoport in intraventricular tumors

Endoport, as a tubular retractor, can effectively help overcome the challenges and fully utilize the advantages of endoscopy. Endoport isolates the brain tissue outside the tubular retractor, providing a safe pathway for surgical instruments inserted freely under direction vision and preventing any damage to the surgical path caused by surgical procedures (7, 22). Moreover, compared with traditional retractor-assisted microsurgery, its tubular structure reduces the risk of sharp damages of sheet retractors caused by traction on brain tissue. The tubular shape of the retractor allows for the even distribution of pressure on the retracted brain tissue, minimizing damage to the greatest extent possible (20). Additionally, endoport allows for movement in multiple angles, enabling full exposure of the tumor without increasing tension on brain tissue. This not only improves the rate of complete tumor resection but also provides better protection for surrounding tissues (12, 22, 23). A study has reported that patients who underwent microsurgical procedures via a trans-cortical approach had a postoperative seizure incidence of 8% and a postoperative paralysis incidence of 12% (24). These complications were largely related to excessive traction on the functional cortical areas. In contrast, none of the cases in our group experienced postoperative complications such as seizures or persistent hemiplegia.

### Application of neuro-navigation in tumors location and inserted position selection

The key to the successful endoscopic tumor surgery lies in the precise localization of the tumor and the selection of the puncture point and direction (16). Thus, the precise location of the tumors on MRI preoperative and intraoperative neuro-navigation are of great importance for brain-deep tumors localization. In a survey of neurosurgeons with experience in endoscopic surgery, the utilization rate of neuro-navigation in the biopsy and resection of intraventricular tumors was found to be 62.4% (25). In our sixteen cases, preoperative MRI and intraoperative neuro-navigation were used in all cases. Besides these, the intraoperative endoscopic ultrasonography and MRI were also reported to be helpful in real-time tumor positioning, facilitating complete tumor removal and protection of nearby structures (26).

The inserted point of Endoport lies on the position of lateral ventricular tumors. For the tumors localized in the frontal horns and the body of the lateral ventricle, we selected trans-frontal cortical approach (middle frontal gyrus). This approach facilitates the exposure of the anterior choroidal artery and is suitable for the resection of tumors in the anterior part of the lateral ventricle. For the tumors localized in the trigone of the lateral ventricle, we selected transtemporal approach. This approach provides a short trajectory and a direct access to the temporal horn and trigone of the lateral ventricle. In our 16 cases, the total resection rate of tumors was 88% through these approaches. Two subtotal resected tumors were glioblastoma and diffused astrocytoma separately. However, due to the risk of damaging the fiber tracts, there is currently ongoing debate regarding the choice between the transcortical and sulcal approaches. Further research is needed to establish reliable conclusions.

### Keep the cerebrospinal fluid circulation unobstructed

Preoperative CSF circulation disturbance was common in patients with lateral ventricular tumors and it was found in 9 cases of our 16 cases. For cases of obstructive hydrocephalus due to the blockage of the foramen of Monro found intraoperative, simultaneous ventriculostomy was performed to establish drainage. Operations within ventricles commonly lead to loss of CSF and collapse of brain tissue (15). In our study, we encountered a case of acute epidural hematoma, which was considered to be caused by rapid collapse of brain tissue resulting from excessive release of cerebrospinal fluid. Thus, the slow release of CSF intraoperatively was necessary to prevent rapid brain tissue collapse and the potential development of epidural hematoma (27, 28). Early postoperative CT scan also acids in early diagnosis. Besides these, it was essential to ensure the patency of the interventricular foramen and avoid blockage by blood clots during surgery. Before completing the procedure, warm saline was perfused to inflate the collapsed brain tissue and reduce intracranial air accumulation. Otherwise, if postoperative external ventricular drainage necessary, the flow velocity and volume needed to be close controlled to prevent the occurrence of ventricular walls adhesion or isolated ventricle (16).

### Postoperative complications and comprehensive treatment

Reported complication rates for resection of intraventricular tumors are between 0% and 30%, including hemorrhage, hydrocephalus, subdural hygroma, deep venous thrombosis, hemiparesis and neurological deficit (**Table 2**) (11–16, 29–32). In our study, 1 case experienced acute epidural hematoma, which was considered due to the following reasons (1): young patient, no adhesion between the pia mater and the dura mater (2), rapid collapse of brain tissue resulting from excessive release of cerebrospinal fluid. Thus, slow intraoperative CSF release is necessary to prevent rapid brain tissue collapse. When Endoport near the ventricular wall, using an electrical coagulation to create a small opening or a ventricular puncture needle could be helpful to release CSF slowly. Besides, tailed cotton strips could be used to plug the fistula to slow down the CSF release if CSF still flows out too quickly. Another two patients developed obstructive hydrocephalus following operation. After corresponding treatment, no permanent sequelae were left.

Additionally, specific pathological types may require further standardized treatment even after discharge. Among the cases in this group, a patient diagnosed with glioblastoma survived for 16 months after the operation, while another patient with diffuse astroglioma achieved long-term survival despite residual tumor. As both tumors originated in the thalamus, preserving the functionality of the structure as much as possible during the surgery led to incomplete tumor resection according to the standard guidelines. Comprehensive treatment is needed for every patient according to separate pathologic diagnosis.

### Limitations

The number of cases in this study is relatively insufficient. There is an inherent bias in the follow-up time, which still needs to be extended. Further studies and long-term follow-up are necessary to validate the long-term therapeutic efficiency and outcomes of this surgical method. Besides, We have limited experience in utilizing other endoscopic-assisted techniques, such as CUSA and LASER. Acquiring more experience in these techniques would undoubtedly contribute to broadening the scope of indications for endoscopic resection under our care.

## Conclusion

Overall, Endoport-assisted neuroendoscopic techniques offer significant advantages in managing lateral ventricular tumors. It effectively utilizes the benefits of close observation, comprehensive exposure, and reduced tissue damage. Therefore, Endoport-assisted neuroendoscopic surgery is suitable for the resection of lateral ventricular tumors. This technique is worthy to be popularized in clinical practice.

## Data availability statement

The original contributions presented in the study are included in the article/supplementary material. Further inquiries can be directed to the corresponding authors.

## Ethics statement

This study was conducted retrospectively from data obtained for clinical purposes. All procedures performed in studies involving human participants were approved by ethics committee at Nanjing Drum Tower Hospital and in accordance with the ethical standards of the institutional and/or national research committee and with the 1964 Helsinki declaration and its later amendments or comparable ethical standards. Informed consent was obtained from all individual participants and their close relatives included in the study. Informed consent was received from participant or legal guardian (in case of patient less than 18 years of age) for participation as well as publication of their data and photographs.

## Author contributions

All authors contributed to the study conception and design. Material preparation, data collection and analysis were performed by CLY and JM. The first draft of the manuscript was written by CLY. JM, CBY and YL participated the surgery and handled the pictures. WJ and HY commented and corrected on previous versions of the manuscript. All authors read and approved the final manuscript. All authors contributed to the article and approved the submitted version.
